# CD40 Pathway and IL-2 Expression Mediate the Differential Outcome of Colorectal Cancer Patients with Different *CSF1R* c.1085 Genotypes

**DOI:** 10.3390/ijms222212565

**Published:** 2021-11-22

**Authors:** Yu-Min Yeh, Peng-Chan Lin, Wu-Chou Su, Meng-Ru Shen

**Affiliations:** 1 Department of Oncology, National Cheng Kung University Hospital, College of Medicine, National Cheng Kung University, Tainan 70456, Taiwan; i5485111@gmail.com (Y.-M.Y.); pengchanlin@gmail.com (P.-C.L.); sunnysu@mail.ncku.edu.tw (W.-C.S.); 2 Department of Obstetrics and Gynecology, National Cheng Kung University Hospital, College of Medicine, National Cheng Kung University, Tainan 70456, Taiwan; 3 Department of Pharmacology, College of Medicine, National Cheng Kung University, Tainan 70456, Taiwan

**Keywords:** colony-stimulating factor 1 receptor, genetic variant, CD40, IL-2 expression, colorectal cancer

## Abstract

Colony-stimulating factor 1 receptor (CSF-1R) acts as the receptor for colony stimulating factor 1, a cytokine that controls the production, differentiation, and function of macrophages. Prior studies showed cancer patients harboring germline *CSF1R* c.1085A>G genetic variant had better survival. Here, primary tumor samples from a stage III colorectal cancer (CRC) cohort were analyzed by a targeted gene expression assay containing 395 immune-related genes to study the immune mechanism underlying the different outcomes. CRC patients with *CSF1R* c.1085 genotype A_G had a better disease-free and overall survival than those with *CSF1R* genotype A_A. Compared to the group of patients without *CSF1R* variant, higher *CD40LG* expression, a surface marker of T cells, was found in the tumor tissues of patients with *CSF1R* c.1085 variant. In parallel with the higher *CD40LG* gene expression, immunofluorescent staining also showed more CD3^+^CD40L^+^ T cell infiltrates in tumors with *CSF1R* c.1085 genotype A_G. Moreover, higher IL-2 expression, known to be regulated by CD40 pathway, was also observed in tumors with *CSF1R* c.1085 genotype A_G than genotype A_A. Higher IL-2 expression generated by the interaction of CD40 ligand and CD40 between T cells and macrophages with *CSF1R* c.1085A>G variant is the potential mechanism explaining the different outcomes.

## 1. Introduction

The immunotherapy targeting cytotoxic T lymphocyte-associated protein 4 (CTLA4) and programmed cell death 1 (PD-1) has recently been demonstrated to be an effective approach to activate immune response and treat a wide range of cancers [1]. However, the response to immunotherapy is not observed in all types of cancers. Moreover, progressive disease eventually develops in most patients who initially respond to immunotherapy. The mechanisms of the primary and secondary resistance are complex and largely determined by the interaction between cancer and immune cells in the microenvironment [2]. The most dominant and import immune infiltrates in the tumor microenvironment are macrophages, which are often termed as tumor-associated macrophages [3]. In majority of cancers, tumor-associated macrophages are pro-tumoral macrophages. These macrophages exhibit pro-tumorigenic effects by suppressing anti-tumor immune response of effector cells, including T cells and NK cells, and promoting tumor proliferation, survival, and metastasis. Furthermore, the production of angiogenic factors and stimulation of angiogenesis by macrophages are demonstrated in a wide range of models [4]. Numerous studies have shown the relationship between tumor-associated macrophages and poor clinical outcome [5]. Therefore, targeting tumor-associated macrophages, especially the tumor-promoting M2-like macrophages, has become a promising therapeutic strategy to improve the efficacy of current immunotherapy in recent years.

**Figure 1:**
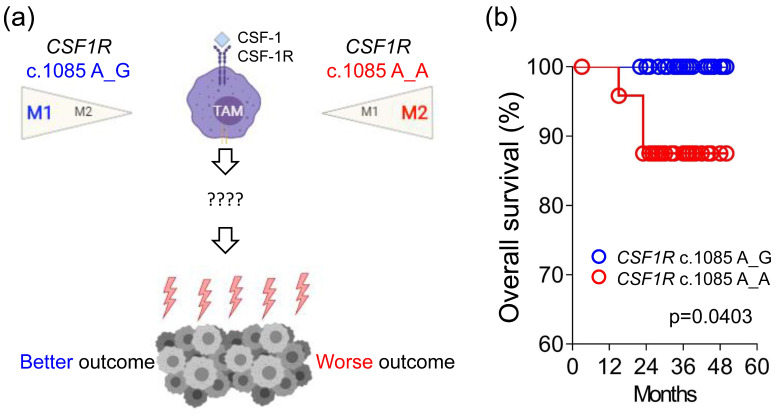
(**a**) Summary of the impacts of *CSF1R* c.1085 genotypes on the macrophage polarization and clinical outcome of cancer patients. The detailed mechanisms mediating the different outcome remain unknown. TAM, tumor-associated macrophage; CSF-1, colony stimulating factor 1; CSF-1R, colony stimulating factor 1 receptor. (**b**) Overall survival of CRC patients with different *CSF1R* c.1085 genotypes. Kaplan–Meier curves of overall survival in CRC patients with *CSF1R* c.1085 genotype A_A and A_G are shown and were compared by log-rank test.

The key pathway regulating the macrophages is colony-stimulating factor 1 receptor (CSF-1R) signaling. CSF-1R, consisting of five extracellular immunoglobulin-like domains, a transmembrane helix, and an intracellular autoinhibitory juxtamembrane domain linked to split tyrosine kinase domains [6], is a class III receptor tyrosine kinase encoded by the *CSF1R* gene [7]. This receptor contains 972 amino acids with a molecular weight of approximately 108 kDa. Our prior study identified an important germline *CSF1R* genetic variant, c.1085A>G, causing the change of amino acid from histidine to arginine in the domain of receptor dimerization [8]. Macrophages with different *CSF1R* c.1085 genotypes displayed different response to CSF-1 stimulation. CSF-1 induced less CSF-1R phosphorylation and endocytosis in macrophages with *CSF1R* c.1085 genotype A_G. The weak CSF-1R phosphorylation was accompanied by less macrophage differentiation and M2 polarization. In addition, cancer patients with *CSF1R* c.1085A>G variant had less tumor-associated macrophages, M2-like macrophages, and VEGF expression in tumor tissues. In parallel with the less M2-like macrophages, the presence of this germline variant was significantly associated with better disease-free survival in patients with colorectal, endometrial, and ovarian cancer. Despite the known relationship between tumor-associated macrophages and poor clinical outcome, the detailed mechanisms mediating different outcome between patients with *CSF1R* c.1085 genotypes A_A and A_G remain unclear (Figure 1a).

Here, we extensively analyzed the expression of genes involved in immune response from 66 primary tumor samples of colorectal cancer (CRC) patients and compared the difference between patients with different *CSF1R* c.1085 genotypes. The results showed CRCs harboring *CSF1R* c.1085 A>G variant had higher *CD40LG* and *IL-2* expression in tumor tissues than those with *CSF1R* c.1085 genotype A_A. More CD3^+^CD40L^+^ T cells and higher IL-2 expression observed in tumors with *CSF1R* c.1085 genotype A_G confirmed the finding of mRNA expression. These results, together with prior reports, suggest higher IL-2 expression generated by the interaction of CD40 ligand and CD40 between helper T cells and macrophages with *CSF1R* c.1085 genotype A_G might be the mechanism explaining why CRC patients with this germline variant had a better clinical outcome.

## 2. Results

### 2.1. The Impact of Germline CSF1R c.1085A>G Genetic Variant on Clinical Outcome

A total of 109 stage III or high-risk stage II CRC patients enrolled in the CIPN study were used to study the potential mechanism mediating the different clinical outcome between patients with *CSF1R* c.1085 genotype A_A and A_G (Figure S1). Our prior study showed CRC patients with *CSF1R* c.1085 A_A had a worse disease-free survival compared to patients with genotype A_G [8]. Until April 2019, median follow-up time for 60 patients with *CSF1R* c.1085 genotype A_A and A_G enrolled in the prospective study was 35 months. The clinical characteristics of these 60 patients are shown in Table 1. There was no significance of difference between these two groups of CRC patients with respect to age, gender, location of primary tumor, and the percentage of *RAS* and *BRAF* mutation, except for the stage. The percentage of stage II disease was higher in the groups of genotype A_A compared to genotype A_G (12% vs. 2.9%). In contrast, patients with *CSF1R* genotype A_A had a lower percentage of stage IIIC disease than those with genotype A_G (4% vs. 25.7%). Three of 25 patients with *CSF1R* c.1085 A_A died, but no death was observed in the group of patients with *CSF1R* c.1085 A_G. Despite the group of *CSF1R* genotype A_A contained less patients with advanced disease, CRC patients with *CSF1R* c.1085 A_A still showed a worse overall survival compared to patients with *CSF1R* genotype A_G (Figure 1b). This result confirmed our prior finding that *CSF1R* c.1085 germline genetic variant had an important impact on clinical outcome. Comprehensively analyzing the tumor–immune interactions in the tumor microenvironment would enable us to understand of the mechanism mediating the different clinical outcome between patients with different *CSF1R* genotypes (Figure 1a).

### 2.2. Differentially Expressed Genes Associated with Immune Response in CRCs with CSF1R c.1085 Genotype A_A and A_G

**Figure 2:**
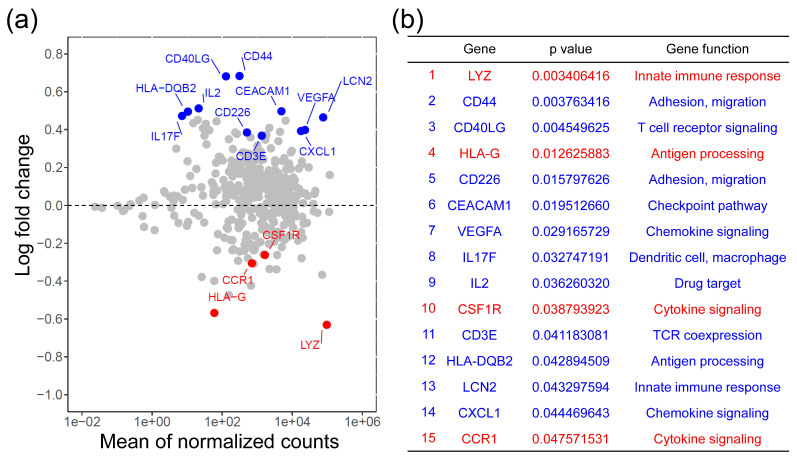
Immune response genes differentially expressed in CRCs with *CSF1R* c.1085 genotype A_A and A_G. (**a**) The MA-plot showing log-fold change against the average of expression levels for each gene between CRCs with *CSF1R* c.1085 genotype A_A and A_G. The *x*-axis indicates the mean of normalized reads for each gene and the *y*-axis indicates the log_2_ fold of change. The red and blue dots represent the fact that the genes had a significantly higher expression level in the groups of genotype A_A and A_G, respectively. (**b**) The list of genes differentially expressed in the groups of *CSF1R* c.1085 genotype A_A and A_G with statistical significance.

To comprehensively study the tumor-immune microenvironment, we performed the Oncomine Immune Response Research Assay, a targeted gene expression assay including 395 genes associated with immune response (Figure S2), on primary CRC tumor samples. After excluding 7 cases with poor library preparation and 18 cases with read less than 500,000, the expression levels of immune response-associated genes could be analyzed in 24 and 42 CRCs with *CSF1R* c.1085 genotype A_A and A_G, respectively (Figure S1). The average and ratio of expression levels for each gene between the groups of *CSF1R* c.1085 genotype A_A and A_G were displayed as MA-plot and shown in Figure 2a. A total of 15 genes were found to be differentially expressed between these two groups of CRCs (Figure 2b). Among these, the expression levels of *LYZ*, *HLA-G*, *CSF1R*, and *CCR1* genes (red dots in Figure 2a) were higher in CRCs with *CSF1R* c.1085 genotype A_A compared to those with genotype A_G. *LYZ* and *HLA-G* were involved in innate immunity and antigen processing, respectively. *CSF1R* and *CCR1* were categorized as genes of cytokine signaling. In contrast, CRCs with *CSF1R* c.1085 genotype A_G had higher expression levels of *CD44*, *CD40LG*, *CD226*, *CEACAM1*, *VEGFA*, *IL17F*, *IL2*, *CD3E*, *HLA-DQB2*, *LCN2*, and *CXCL1* gene (blue dots in Figure 2a) than the group of genotype A_A. *CD44* and *CD226* were genes associated with cell adhesion and migration. *CD40LG*, encoding the protein CD40 ligand (CD40L), was involved in T cell receptor signaling. Other genes were involved in a variety of immune processes, including checkpoint pathway, chemokine signaling, T cell receptor coexpression, antigen processing, and innate immunity.

### 2.3. CRCs with CSF1R c.1085 A_G Were Associated with Higher Number of CD40L^+^ T Cells

**Figure 3:**
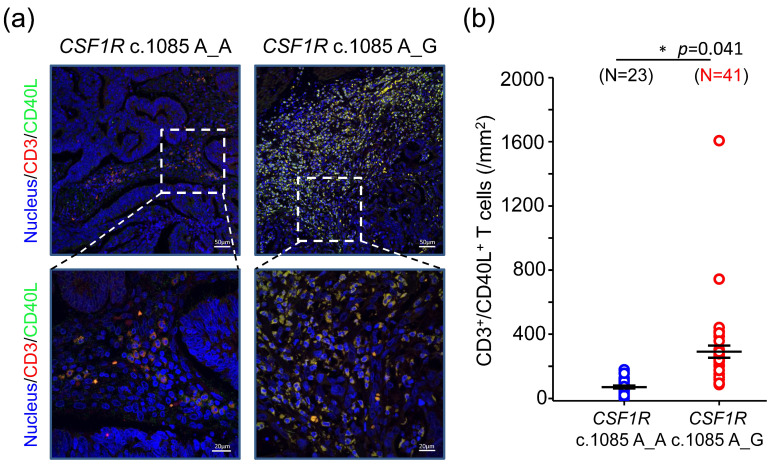
*CSF1R* c.1085A>G genetic variant was associated with the distribution of CD40L^+^ T cells in CRCs. (**a**) Representative confocal images co-stained with anti-CD3 (for T cells) and anti-CD40L (for CD40 ligand) in primary CRC tumor specimens are shown. (**b**) In each sample, the region of interest was randomly selected from 5 fields of tumor microenvironment. Each field was 1.2 × 1.2 mm^2^. The number of CD3^+^CD40L^+^ cells was quantitated automatically by TissueQuest software and compared between the group of *CSF1R* c.1085 A_A and A_G. *, *p* < 0.05.

To investigate the potential mechanism mediating the better clinical outcome in CRCs with *CSF1R* c.1085 genotype A_G, we selected the genes with higher expression level in the group of genotype A_G for further study. Among these genes, *CD44* is the top one gene, followed by *CD40LG*. CD44 is a cell-surface glycoprotein that has been known to be involved in cell–cell interaction, cell adhesion, and migration. Prior studies showed that CD44 plays an important role in macrophage recruitment [9]. CD44-deficient macrophages showed reduced ability of adhesion and migration in vitro and in vivo. In the group of CRCs with *CSF1R* c.1085 genotype A_G, *CD44* expression level was higher compared to the group of genotype A_A. However, when we used CD68 to stain and quantify the macrophages in CRC tumor samples, CRCs with *CSF1R* c.1085 genotype A_G did not contain higher number of macrophages than those with *CSF1R* genotype A_A [8], suggesting *CD44* expression levels did not show a positive correlation with the number of macrophages in tumor tissue. The protein encoded by *CD40LG* is CD40 ligand, a protein mainly expressed on the surface of T cell. The interaction of CD40 and CD40L between macrophages and T cells had been shown to play important roles in enhancing adaptive T cell response through the activation of dendritic cells/macrophages and induction of IL-2 [10]. To study whether CD40L-mediated immune response is a potential mechanism explaining the different clinical outcome between CRCs with different *CSF1R* genotypes, we examined the number of CD40L^+^ T cells in primary CRC tumor samples. Anti-CD3 and anti-CD40L antibodies were used to stain T cells and the expression of CD40L, respectively. As shown in Figure 3a,b, the number of CD3^+^CD40L^+^ T cells was significantly higher in the tumors with *CSF1R* c.1085 genotype A_G.

### 2.4. IL-2 Expression Was Higher in CRCs with CSF1R c.1085 Genotype A_G than Genotype A_A

**Figure 4:**
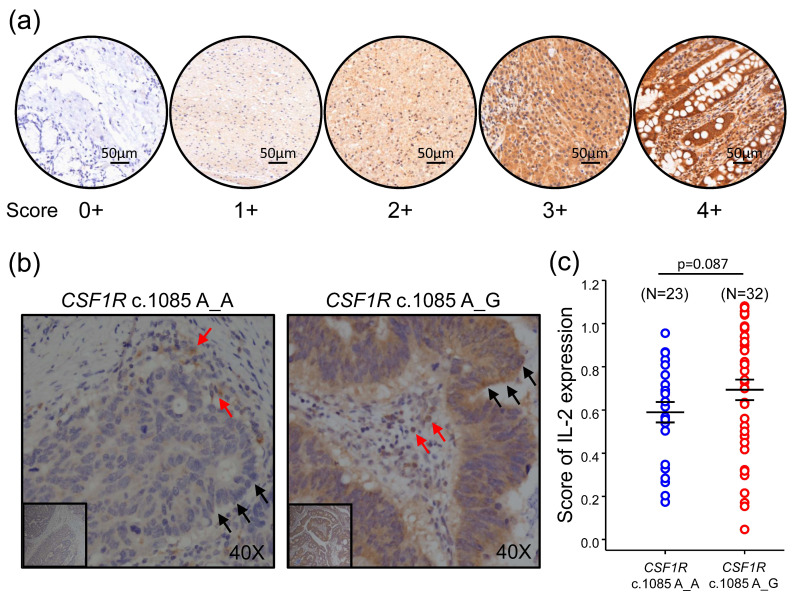
CRC with *CSF1R* c.1085 genotype A_G had higher IL-2 expression. (**a**) Representative images of CRC tumor specimen staining with IL-2 antibody. The IL-2 staining was scored as 1, 2, 3, and 4, according to the intensity. (**b**) Representative images of IL-2 immunohistochemical staining in CRCs with *CSF1R* c.1085 genotype A_A and A_G. Black and red arrows indicate the tumor and immune cells, respectively. (**c**) Whole tissue was scanned with TissueFAXS Plus. The IL-2 expression was determined in 5 randomly selected tumor fields by HistoQuest. Each field was 1.2 × 1.2 mm^2^. The IL-2 expression level was compared between the group of *CSF1R* c.1085 genotype A_A and A_G. Solid lines, mean +/− SEM. Parentheses indicate case number in each group.

The engagement of CD40 and CD40L had been shown to induce the expression of IL-2, which is important for cytotoxic T cell priming [11]. The data of Immune Response Assay also showed higher *IL-2* expression level in CRCs with *CSF1R* c.1085 genotype A_G than genotype A_A (Figure 2b). These results imply the CD40-CD40L signaling, and IL-2 expression might play a role in CRCs and contribute to the different clinical outcome between groups with *CSF1R* genotypes A_A and A_G. To confirm the finding of different IL2 expression in the groups of *CSF1R* c.1085 genotype A_A and A_G, we used anti-IL-2 specific antibody to stain IL-2 in CRC tumor samples. As shown in Figure 4a, a heterogeneous staining pattern with the intensity varying from weak to intense was found in these CRC tumor samples. In addition, the immunoreactivity of IL-2 was observed in both tumor (black arrows) and immune cells (red arrows). The IL-2 staining was scored as 1, 2, 3, or 4 according to the intensity. We calculated the score of IL2 expression by multiplying the percentage of positive areas by the intensity. Compared to CRCs with *CSF1R* c.1085 genotype A_A, a trend showing higher IL-2 expression in CRC with *CSF1R* c.1085 genotype A_G was observed (Figure 4b,c). This result was in agreement with the finding in mRNA expression.

## 3. Discussion

Immunotherapy has shown a great advance in cancer treatment. However, primary and secondary resistances are still important clinical issues to be resolved. Better understanding of the tumor immune microenvironment is essential to overcome the resistance and establish efficient cancer immunotherapies. By analyzing the clinical outcome and tumor immune microenvironment from 109 stage III or high-risk stage II CRC patients, this study identified several important findings: (i) CRC patients with *CSF1R* c.1085 genotype A_A had a worse overall survival than patients with *CSF1R* genotype A_G. (ii) Higher *CD40LG* and *IL-2* expression was observed in tumor tissues with genotype A_G than those with genotype A_A. (iii) Tumors with *CSF1R* c.1085 genotype A_G contained more CD3^+^CD40L^+^ T cells. Moreover, higher IL-2 expression was also observed in tumors with *CSF1R* c.1085 genotype A_G compared to genotype A_A. These confirmed the finding of mRNA expression. The CD40 pathway has been demonstrated to be a key regulator of cytokine production, including IL-2, and anti-tumor immune response [11]. Taking the results of prior studies and our studies together, we found that higher IL-2 expression generated by the interaction of CD40 ligand and CD40 between helper T cells and macrophages with *CSF1R* c.1085 genotype A_G is the potential mechanism explaining why CRC patients with this germline variant had a better clinical outcome.

The clinical impacts of *CSF1R* c.1085 genotype had been investigated in several studies [12,13]. However, the association between *CSF1R* c.1085 genotype and clinical outcome of cancer patients was rarely reported. Our prior study demonstrated germline *CSF1R* c.1085A>G variant was associated with disease-free survival in stage III or high-risk stage II CRC patients [8]. In addition, a better overall survival and a trend showing better outcome in the variant group were observed in patients with endometrial cancer and ovarian cancer, respectively. With a longer follow-up period and more enrollment for the CRC cohort, we demonstrated patients with *CSF1R* c.1085 A_G had a better overall survival compared to patients with *CSF1R* genotype A_A in this study, consistent with our prior findings. Only one genome-wide association study using a Korean non-small cell lung cancer cohort reported the impact of *CSF1R* c.1085A>G variant (rs10079250) on clinical outcome. In agreement with our findings, Yoo et al. showed *CSF1R* rs10079250 A>G was associated with a better disease-free survival in never-smoking females [13]. These data suggested *CSF1R* c.1085A>G variant had an impact on clinical outcome of cancer patients.

A more understanding of the mechanism mediating the different outcome of patients with and without *CSF1R* c.1085A>G variant may help to develop new therapeutic strategies for patients with different genotypes. In the present study, higher *CD40LG* expression was observed in tumor tissues with *CSF1R* c.1085 genotype A_G compared to those with genotype A_A. The protein encoded by *CD40LG* is CD40 ligand (CD40L) expressed on the surface of T cell. In parallel with the gene expression, higher CD3^+^CD40L^+^ T cells infiltrates were also noted in tumors with *CSF1R* c.1085 genotype A_G. CD40 is the receptor found on macrophages. Our gene expression data showed *CD40* expression was slightly higher in CRCs with *CSF1R* genotype A_G compared to genotype A_A (log_2_ fold of change: 0.04849 and *p* = 0.772221), and our prior study demonstrated the number of macrophages was significantly higher in tumor tissues with the genetic background of *CSF1R* c.1085 genotype A_A compared to genotype A_G [8]. The higher CD40 expression and lower macrophages in CRCs with *CSF1R* c.1085 genotype A_G suggest the level of CD40 expression might be higher in macrophages with *CSF1R* c.1085 genotype A_G than genotype A_A. Further studies are still needed to confirm this hypothesis. The interaction between CD40 on macrophages and CD40L on lymphocytes had been known to induce intracellular signaling pathways and subsequent IL-2 expression, which is important for cytotoxic T cell priming. Our study also demonstrated CRC tumors with *CSF1R* c.1085 genotype A_G had higher IL-2 gene and protein expression. Therefore, higher IL-2 expression generated by the interaction of CD40 ligand and CD40 between helper T cells and macrophages with *CSF1R* c.1085 genotype A_G was the potential mechanism explaining why CRC patients with this germline variant had a better clinical outcome. These findings provide a rationale to test the utility of IL-2 in CRC patients with *CSF1R* c.1085 genotype A_A.

CRCs with *CSF1R* c.1085 genotype A_G had higher IL-2 gene expression than those with genotype A_A; however, the difference in IL-2 protein expression was not statistically significant but trending. This may be explained by the case number and different stage of the lesions distributed in different groups of *CSF1R* genotyping. Moreover, the IL-2 gene and protein expression measured in this study were the total IL-2 expressed by both tumor and non-tumor cells in the tumor immune microenvironment. It is not easy to specifically determine IL-2 level generated by immune cells in tumor tissues. Further studies, such as co-culture of CD4^+^ T cells with macrophages with and without *CSF1R* c.1085A>G variant, will be needed to confirm this hypothesis.

In addition to the differential *CD40LG* and *IL-2* expression shown in this study, our prior work demonstrated the impact of germline *CSF1R* c.1085A>G variant on CSF-1R signaling and macrophage functions [8]. Macrophages with *CSF1R* c.1085A>G variant displayed a poor response to CSF-1 stimulation. In addition, patients harboring this variant had fewer total macrophages and M2-like macrophages in tumor tissues. Interestingly, the gene expression data also showed higher *CSF1R* mRNA expression in CRCs with *CSF1R* c.1085 genotype A_A compared to genotype A_G. It is very likely that CRCs with *CSF1R* c.1085 genotype A_A had higher CSF-1R protein expression than those with genotype A_G. CSF-1R is primarily expressed on macrophages, and it has been known that CSF-1R signaling is important to main macrophages in an M2-like state. Higher proportion of M2 macrophages associated with advanced stage and poor clinical outcome has been reported in many different types of cancer [14]. The underlying mechanism mediating this correlation has also been extensively studied in vitro and in vivo [15]. Lower CSF-1R expression, less tumor-associated macrophages, and less M2-like macrophages in tumors with *CSF1R* genotype A_G also provide an explanation why patients with this germline variant had better clinical outcome. The CSF-1R expression on macrophages and the underlying mechanism mediating higher M2-like macrophage and less CD3^+^CD40L^+^ T cell infiltrates in tumors with *CSF1R* c.1085 genotype A_G is worth further exploration.

**Figure 5:**
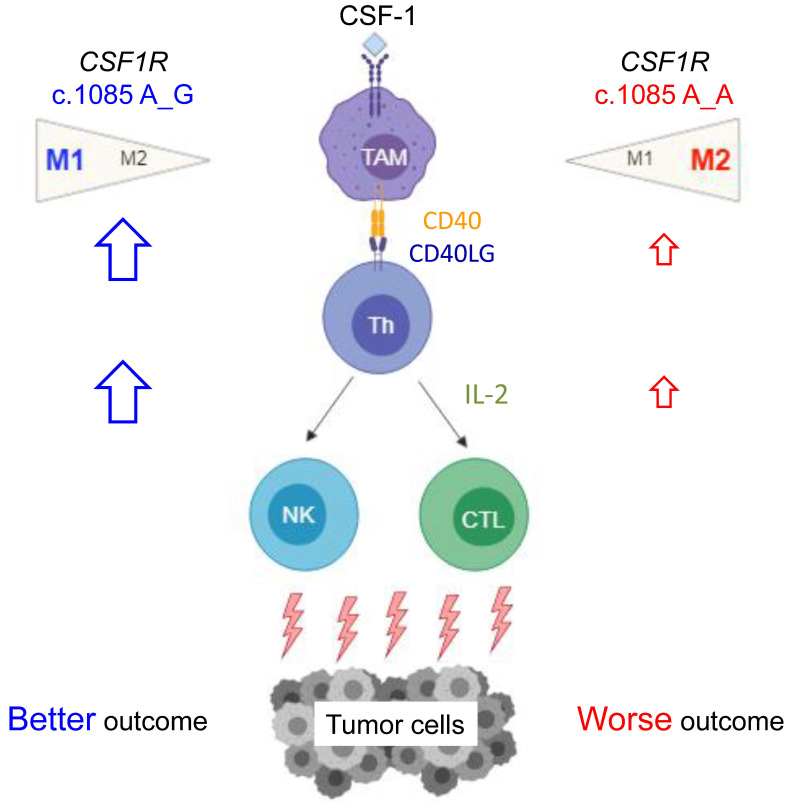
*CSF1R* c.1085 genotype regulated tumor immunity and affected clinical outcome. Macrophages with different *CSF1R* c.1085 genotypes responded to the stimulation of CSF-1 differentially. CSF-1 induced a prominent activation of CSF-1R, leading to an M2-like dominant polarization in macrophages with *CSF1R* c.1085 genotype A_A. CRC patients with *CSF1R* c.1085 genotype A_A had worse clinical outcome, including shorter disease-free survival and overall survival. In addition to the more M2-like macrophages, lower IL-2 expression generated by the interaction of CD40 ligand and CD40 between helper T cells and macrophages with *CSF1R* c.1085 genotype A_A also provide a mechanism explaining why CRC patients without this germline variant had a worse clinical outcome. TAM, tumor-associated macrophage; Th, T helper cell; NK, nature killer cell; CTL, cytotoxic T lymphocyte.

Taking this and our prior study together, we find that germline *CSF1R* c.1085A>G variant had a great impact on macrophage functions, tumor immune microenvironment, and clinical outcome (summarized in Figure 5). Macrophages with *CSF1R* c.1085A>G variant displayed a poor response to CSF-1 stimulation in terms of CSF-1R phosphorylation, macrophage differentiation, and M2 polarization. CRCs with *CSF1R* c.1085 genotype A_G contained less M2-like macrophage infiltrates in tumor tissues. In addition to less M2 distribution, higher *CD40LG* expression and CD3^+^CD40L^+^ T cells infiltrates were also observed in CRCs with *CSF1R* c.1085A>G variant and accompanied by higher IL-2 expression, which is important in activation of nature killer (NK) cells and cytotoxic lymphocytes. These findings explained how *CSF1R* germline c.1085 genetic variant impacted the tumor microenvironment and why patients with *CSF1R* c.1085 genotype A_G had a better clinical outcome than those with genotype A_A. Since IL-2 had been applied in cancer immunotherapy clinically, it is worth testing the utility of IL-2 in CRC patients with *CSF1R* c.1085 genotype A_A.

## 4. Materials and Methods

### 4.1. Database Used for Analysis

The database generated from a retrospective pilot study and an ongoing prospective study evaluating the association of germline genetic variants and chemotherapy-induced peripheral neuropathy (CIPN) [16] was used for analysis in this study. These 2 studies were approved by the institutional review board (A-ER-103-395 and A-ER-1040153) of National Cheng Kung University Hospital (NCKUH), Tainan, Taiwan, and conducted in accordance with the Declaration of Helsinki. Written informed consent was provided by all patients. The information of these 2 CIPN studies was described in detail in the prior study [8]. In brief, stage III or high-risk stage II CRC patients receiving standard surgical resection followed by adjuvant chemotherapy with mFOLFOX6 were enrolled. Whole genome sequencing was performed on DNA generated from peripheral blood to determine germline genetic variants. Until July 2017, 109 CRC patients, including 37 patients in the pilot study and 72 patients in the prospective study, were enrolled. Primary tumor samples of these patients were used to investigate the expression of immune response gene expression, and 72 patients enrolled in the prospective study were used to study the impact of germline *CSF1R* c.1085 genetic variant on clinical outcome.

### 4.2. RNA Sequencing

The RNA was isolated from paraformaldehyde-fixed paraffin-embedded (FFPE) primary CRC tumor tissue using RecoverAll Total Nucleic Acid Isolation Kit (Thermo Fisher Scientific, Waltham, MA, USA). RNA concentration was determined by Invitrogen™ Qubit™ Fluorometer with the Qubit™ RNA High Sensitivity Assay (Thermo Fisher Scientific). SuperScript™ IV VILO™ Master Mix Kit was used to perform reverse transcription with 20ng RNA. Immune Response libraries were prepared using the Ion AmpliSeq™ Kit for Chef DL8 with the Ion Chef™ System. The conditions of library preparation on the Ion Chef™ System was performed according to the Oncomine™ Immune Response Research Assay user guide (pub. no. MAN0015867). Template preparation, chip loading, and sequencing were carried out on the Ion Chef™ System and Ion S5 XL sequencing system with Ion 510 and Ion 520 and Ion 530 Kit-Chef Kit (Thermo Fisher Scientific, Waltham, MA, USA). The Ion Chef™ and Ion S5 XL sequencing system (Thermo Fisher Scientific, Waltham, MA, USA) were used as described in the User Guide: Ion 510 and Ion 520 and Ion 530 Chef Kit—Instructions for automated template preparation, chip loading, and sequencing (pub. no. MAN0016854). The raw gene expression data were preprocessed using Torrent Suite (Thermo Fisher Scientific, Waltham, MA, USA) and analyzed using DESeq2 package in R. Data normalization was performed via function DESeq. Candidate genes that were differentially expressed between the groups of *CSF1R* c.1085 genotype A_A and A_G were selected for further study.

### 4.3. Chemicals and Antibodies

The antibodies used for immunofluorescent staining were mouse anti-CD3 (Thermo Fisher Scientific Cat# 14-0037-82, RRID: AB_467057), rabbit anti-CD40L (Abcam Cat# ab2391, RRID: AB_303034), and Hoechst 33258 (Thermo Fisher Scientific Cat# H3569, RRID: AB_2651133). Alexa Fluor 594- (Thermo Fisher Scientific, Waltham, MA, USA, Cat# A-21203, RRID: AB_2535789) and Alexa 488-conjugated secondary antibodies (Thermo Fisher Scientific, Waltham, MA, USA, Cat# A-11008, RRID: AB_143165) were purchased from Thermo Fisher Scientific Inc (Waltham, MA, USA). Rabbit anti-IL-2 polyclonal antibody (Bioss, Woburn, MA, USA, Cat# bs-0605R, RRID: AB_10856306) was the primary antibody used for immunohistochemical staining.

### 4.4. Immunofluorescent Staining, Confocal Images, and Tissue Scanning

Primary tumor specimens of CRC patients enrolled in CIPN studies were used for immunofluorescent staining. We used anti-CD3 and anti-CD40L to stain T cells and CD40 ligand, respectively. Alexa Fluor 594-conjugated secondary antibody was used for CD3, and Alexa 488-conjugated secondary antibody was used for CD40L. Tissue was co-stained with Hoechst to detect the nucleus. Whole tissue was scanned automatically using a TissueFAXS PLUS microscope (TissueGnostics, Vienna, Austria). In each sample, the region of interest (ROI) was randomly selected from 5 fields of the tumor microenvironment. Each field was 1.2 × 1.2 mm^2^. The number of CD3^+^CD40L^+^ cells was quantitated automatically by TissueQuest analysis software (RRID: SCR_014822).

### 4.5. Immunohistochemical Staining

Immunohistochemical (IHC) staining was performed on 4 µm thick formalin-fixed paraffin-embedded sections. We used rabbit anti-human IL-2 polyclonal antibody (1:100 dilution) as the primary antibody. The procedures were conducted with the Bond-Max Automated IHC stainer (Leica Biosystems, Lincolnshire, IL, USA) according to the following protocol. Tissues were deparaffinized with xylene and pre-treated with the Epitope Retrieval Solution 2 (EDTA buffer, pH 9·0) at 100 °C for 20 min. After deparaffinization, the tissues were incubated with primary antibody at room temperature for 30 min. Subsequently, tissues were incubated with polymer at room temperature for 8 min using the Bond Polymer Refine Detection Kit (Leica Biosystems, Lincolnshire, IL, USA) and then developed with 3,3′-diaminobenzidine chromogen for 10 min. Counterstaining was carried out with hematoxylin. In each sample, the region of interest (ROI) was randomly selected from 5 fields of the tumor microenvironment to determine the IL-2 expression level. Each field was 1.2 × 1.2 mm^2^. The staining intensity of IL-2 was categorized into 5 categories as follows: no staining (0+), faint (1+), weak (2+), moderate (3+), and intense (4+). The percentage of positive staining area for each intensity was analyzed by HistoQuest analysis software (RRID: SCR_014822). The IL-2 expression level was scored by multiplying the percentage of positive areas by the intensity.

### 4.6. Statistical Analysis

Kaplan–Meier survival analysis was used to estimate the survival, and the log-rank test was used to compare the difference between groups. All values were presented as mean +/− SEM. Unpaired *t*-test was used to compare the difference between groups. A *p*-value < 0.05 was considered statistically significant.

## 5. Conclusions

Higher IL-2 expression generated by the interaction of CD40 ligand and CD40 between T cells and macrophages with *CSF1R* c.1085 genotype A_G is the potential mechanism explaining why CRC patients with this germline variant had a better clinical outcome.

## Figures and Tables

**Table 1:** Clinical characteristics of 60 CRC patients.

Clinical Characteristic	Total N (%)	*CSF1R* c.1085 A_A N (%)	*CSF1R* c.1085 A_G N (%)	*p*-Value *
Age				0.1740
Median (range)	58.5 (29–78)	69 (39–76)	57 (29–78)	
Gender				0.1239
Male	31 (51.7)	16 (64.0)	15 (42.9)	
Female	29 (48.3)	9 (36.0)	20 (57.1)	
Stage				0.0470
II	4 (6.7)	3 (12.0)	1 (2.9)	
IIIA	8 (13.3)	2 (8.0)	6 (17.1)	
IIIB	38 (63.3)	19 (76.0)	19 (54.3)	
IIIC	10 (16.7)	1 (4.0)	9 (25.7)	
Location				0.3926
Right	15 (25.0)	5 (20.0)	10 (28.6)	
Left	44 (73.3)	19 (76.0)	25 (71.4)	
Right and left	1 (1.7)	1 (4.0)	0 (0)	
*RAS* status				0.6031
Wild-type	31 (51.7)	11 (44.0)	20 (57.1)	
*KRAS* mutation	27 (45.0)	13 (52.0)	14 (40.0)	
*NRAS* mutation	2 (3.3)	1 (4.0)	1 (2.9)	
*BRAF*				0.6339
Wild-type	56 (93.3)	24 (86.0)	32 (91.4)	
Mutant	4 (6.7)	1 (4.0)	3 (8.6)	

* Continuous variable was compared by unpaired *t*-test. Categorical variables were compared by Fisher’s exact test.

## Data Availability

The data presented in this study are available on request from the corresponding author.

## Supplementary Materials

The following are available online at https://www.mdpi.com/article/10.3390/ijms222212565/s1.

## References

[1] Rotte A. (2019). Combination of CTLA-4 and PD-1 blockers for treatment of cancer. J. Exp. Clin. Cancer Res..

[2] Sharma P., Hu-Lieskovan S., Wargo J.A., Ribas A. (2017). Primary, Adaptive, and Acquired Resistance to Cancer Immunotherapy. Cell.

[3] Chen F., Zhuang X., Lin L., Yu P., Wang Y., Shi Y., Hu G., Sun Y. (2015). New horizons in tumor microenvironment biology: Challenges and opportunities. BMC Med..

[4] Nucera S., Biziato D., De Palma M. (2011). The interplay between macrophages and angiogenesis in development, tissue injury and regeneration. Int. J. Dev. Biol..

[5] Edin S., Wikberg M.L., Dahlin A.M., Rutegard J., Oberg A., Oldenborg P.A., Palmqvist R. (2012). The distribution of macrophages with a M1 or M2 phenotype in relation to prognosis and the molecular characteristics of colorectal cancer. PLoS ONE.

[6] Verstraete K., Savvides S.N. (2012). Extracellular assembly and activation principles of oncogenic class III receptor tyrosine kinases. Nat. Rev. Cancer.

[7] Sherr C.J., Rettenmier C.W., Sacca R., Roussel M.F., Look A.T., Stanley E.R. (1985). The c-fms proto-oncogene product is related to the receptor for the mononuclear phagocyte growth factor, CSF-1. Cell.

[8] Yeh Y.M., Hsu S.J., Lin P.C., Hsu K.F., Wu P.Y., Su W.C., Chang J.Y., Shen M.R. (2017). The c.1085A>G Genetic Variant of *CSF1R* Gene Regulates Tumor Immunity by Altering the Proliferation, Polarization, and Function of Macrophages. Clin. Cancer Res..

[9] Hollingsworth J.W., Li Z., Brass D.M., Garantziotis S., Timberlake S.H., Kim A., Hossain I., Savani R.C., Schwartz D.A. (2007). CD44 regulates macrophage recruitment to the lung in lipopolysaccharide-induced airway disease. Am. J. Respir Cell Mol. Biol..

[10] Daoussis D., Andonopoulos A.P., Liossis S.N. (2004). Targeting CD40L: A promising therapeutic approach. Clin. Diagn Lab. Immunol..

[11] Loskog A.S., Eliopoulos A.G. (2009). The Janus faces of CD40 in cancer. Semin Immunol..

[12] Egan K.M., Thompson R.C., Nabors L.B., Olson J.J., Brat D.J., Larocca R.V., Steven B., Paul L.M., Melissa H.M., James E.B. (2011). Cancer susceptibility variants and the risk of adult glioma in a US case-control study. J. Neurooncol..

[13] Yoo S.S., Kang H.G., Choi J.E., Do S.K., Lee W.K., Choi S.H., Lee S.Y., Lee J., Cha S., Kim C.H. (2017). Effects of polymorphisms identified in genome-wide association studies of never-smoking females on the prognosis of non-small cell lung cancer. Cancer Genet..

[14] Mantovani A., Sozzani S., Locati M., Allavena P., Sica A. (2002). Macrophage polarization: Tumor-associated macrophages as a paradigm for polarized M2 mononuclear phagocytes. Trends Immunol..

[15] Ruffell B., Coussens L.M. (2015). Macrophages and therapeutic resistance in cancer. Cancer Cell..

[16] ClinicalTrials.Gov: To Study the Individual Variants of Chemotherapy-Induced Neurotoxicity. https://clinicaltrials.gov/ct2/show/NCT02481336?term=meng-ru+shen&rank=1.

